# The Janus face of L-arginine supplementation in cardiovascular health and aging exploration of underlying mechanisms

**DOI:** 10.3389/fragi.2026.1880662

**Published:** 2026-07-16

**Authors:** Zhihong Yang, Xiu-Fen Ming, Duilio M. Potenza

**Affiliations:** Laboratory of Cardiovascular and Aging Research, Department of Endocrinology, Metabolism, and Cardiovascular System, Faculty of Science and Medicine, University of Fribourg, Fribourg, Switzerland

**Keywords:** aging, arginase, cardiovascular disease, eNOS, mTORC1, oxidative stress

## Abstract

Since the discovery of L-arginine-nitric oxide (NO) pathway in control of cardiovascular function in 1980s, endothelial dysfunction characterized by decreased endothelial NO bioavailability is recognized as a critical hallmark of cardiovascular disease (CVD) and aging, L-arginine supplementation has been proposed as a potential therapeutic intervention primarily due to its role as the sole substrate for endothelial NO-synthase (eNOS). Although many experimental and clinical studies show improvement of endothelial functions by L-arginine supplementation, the beneficial effects could not be translated into the improvement of hard endpoint outcome. Even harmful effects such as worsening of endothelial function, cardiac function, and increased mortality in patients with advanced CVD are reported in the randomized clinical trials. Despite of the reported adverse effects in clinical trials, L-arginine intake is considered safe and is still widely used in our society to boost muscle mass in healthy and aged populations. While we believe that chronic L-arginine supplementation shall be carefully implemented in clinical settings or shall be avoided in patients with advanced CVD and aged population, future studies shall focus on investigation of the underlying mechanisms of the adverse effects of L-arginine supplementation. This is important not only for L-arginine supplementation but also for other amino acid supplementation aimed to improve cardiovascular health, particularly in the elderly population.

## 1 Introduction

**FIGURE 1:**
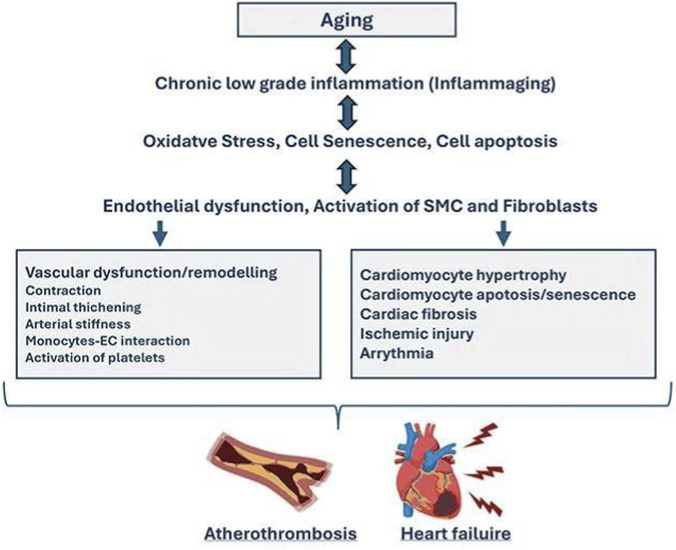
Aging and cardiovascular diseases. Interaction of chronic low-grade inflammation in aging (inflammaging), oxidative stress, cell senescence, apoptosis, endothelial dysfunction, and activation of smooth muscle cells and fibroblasts participates in vascular dysfunction, structural remodelling, and cardiac phenotype changes, which ultimately leads to atherothrombosis and heart failure.

Aging is the most prominent risk factor for cardiovascular disease (CVD), surpassing traditional risk factors such as hypertension, diabetes, and smoking in its impact on disease incidence and mortality (Ahmed et al., 2024). Aging is associated with a group of significant maladaptive structural and functional alterations of heart and blood vessels, leading to accelerated pathogenesis of vascular diseases and heart failure. At the organ levels, cardiovascular aging is manifested with arterial stiffening, endothelial dysfunction, vascular neointimal formation and lumen narrowing, increased vascular inflammation, cardiac remodelling including cardiomyocyte hypertrophy, apoptosis, inflammation, and fibrosis. All these structural and functional changes directly contribute to the pathogenesis of atherosclerosis, stroke, myocardial infarction, and heart failure (Hamczyk et al., 2020; Forman et al., 2023; Ahmed et al., 2024) (Figure 1).

At the cellular and molecular levels, twelve mechanisms or aging hallmarks are proposed to play an orchestrated role in organismal aging (Lopez-Otin et al., 2023). These mechanisms are also shown to be involved in cardiovascular aging and age-accelerated cardiovascular diseases, including low-grade chronic inflammation (Inflammaging), genomic mutation, telomere attrition, epigenetic alterations, altered proteostasis, deregulated nutrition sensing, mitochondrial dysfunction, stem cell exhaustion, cellular senescence, etc., (Donato et al., 2018; Zhong et al., 2026). Recently, multiple large-scale human studies using proteomic, multi-omics, and imaging-based approaches have demonstrated that different organs do not age at the same rate within the same individuals, which is associated with increased risk of organ-specific diseases and mortality (Nie et al., 2022; Oh et al., 2023; Tian et al., 2023; Goeminne et al., 2025; Wang et al., 2025; Consortium et al., 2026). These studies implicate that biological aging, reflected in cellular and molecular hallmarks, is more relevant than chronological aging in organ failure or disease development. The American Heart Association (AHA) highlights that biologic aging more accurately predicts CVD burden and progression than chronological age alone, underscoring the centrality of aging in cardiovascular risk stratification and management (Damluji et al., 2025). Early identification and intervention targeting the mechanisms of cardiovascular aging shall be critical for reducing CVD morbidity and mortality in the aging population (Forman et al., 2023).

## 2 Dysfunctional L-arginine/NO pathway in cardiovascular aging

Since the discovery of endothelial nitric oxide (NO) as a vasodilator by Robert Furchgott in 1980 (Furchgott and Zawadzki, 1980), endothelial dysfunction in cardiovascular aging is well known as one of the earliest mechanisms discovered that links aging to cardiovascular disease. The endothelial dysfunction with a central role of dysfunctional L-arginine/NO pathway, is characterized by impaired vasodilatory, antithrombotic, and anti-inflammatory properties of the endothelial cells. This aspect is deeply reviewed and updated currently, and readers are asked to refer to the review article written by Adhikari et al. (2025). In the present short article, we will focus on analysing the effects (beneficial and detrimental) of L-arginine supplementation in cardiovascular aging and health and the possible underlying mechanisms.

The vasoprotective NO is synthesized from the precursor semi-essential amino acid L-arginine by endothelial NO synthase (eNOS), requiring the cofactor tetrahydrobiopterin (BH4) (Crabtree et al., 2009; Tejero and Stuehr, 2013; Wu et al., 2021). Bioavailable NO declines with aging because of decreased production and increased degradation. Although eNOS expression in vascular endothelial cells is not reduced with aging, and is often upregulated, its enzymatic function is compromised due to “eNOS uncoupling”, a condition characterized by decreased NO production and increased superoxide (O_2_^−^) generation (Rajapakse et al., 2011; El Assar et al., 2012; Yepuri et al., 2012; Donato et al., 2015; Paneni et al., 2017; Donato et al., 2018). Oxidative stress and chronic low-grade inflammation, both hallmarks of vascular aging, accelerate NO degradation. Increased O_2_^−^ reacts with NO to form peroxynitrite (ONOO^−^), a potent oxidant that further impairs endothelial function (Pacher et al., 2007; Griendling et al., 2016; Prolo et al., 2024). The net result is diminished NO-mediated vasorelaxation, increased vascular stiffness, and a pro-atherogenic environment, all of which contribute to the elevated cardiovascular risk seen in older adults. Therefore, the dysfunctional L-arginine/NO metabolism plays a key role in age-related eNOS-uncoupling.

### 2.1 Mechanisms of eNOS-uncoupling

**FIGURE 2:**
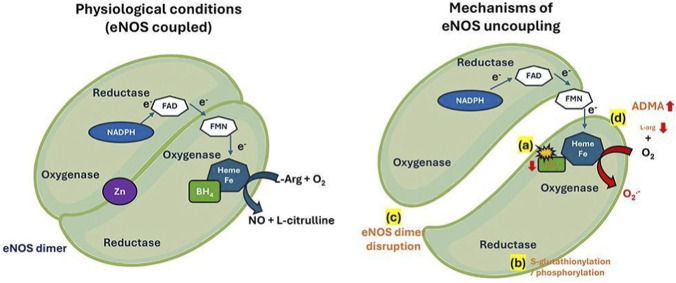
Physiological eNOS function and mechanisms of eNOS-uncoupling. Left panel: under physiological conditions, eNOS functions as a dimer composed of a reductase domain (NADPH → FAD → FMN electron transfer) and an oxygenase domain (heme-Fe center), where electrons and oxygen convert L-arginine to nitric oxide (NO) and L-citrulline. Right panel: Illustration of four major mechanisms promote eNOS-uncoupling: **(a)** depletion of BH_4_ and its oxidation to BH_2_, **(b)** post-translational modifications such as S-glutathionylation and phosphorylation, **(c)** disruption of eNOS dimerization into monomers, and **(d)** L-arginine deficiency together with excess asymmetric dimethylarginine (ADMA). These alterations shift eNOS from NO production toward superoxide (O_2_^−^) generation.

eNOS uncoupling refers to a pathological state in which electrons flowing from NADPH through FAD and FMN to the heme centre in eNOS are no longer properly coupled to L-arginine oxidation, these electrons are instead misdirected to molecular oxygen (O_2_), resulting in superoxide (O_2_•^−^) generation rather than NO production. Under pathological conditions, the proportion of uncoupled eNOS increases, leading to reduced NO bioavailability and increased superoxide (O_2_•^−^) production, contributing to endothelial dysfunction and vascular disease (Yang et al., 2009; Yepuri et al., 2012). The principal mechanisms of eNOS-uncoupling have been shown to be attributable to the following as discussed here briefly (Figure 2). (a) Tetrahydrobiopterin (BH_4_) deficiency or oxidation. BH_4_ is an essential cofactor for eNOS, it ensures electrons delivered to heme are used for L-arginine oxidation (Crabtree et al., 2009). If BH_4_ is depleted or oxidized to BH_2_, often due to increased ROS such as O_2_^−^ and peroxynitrite, electrons escape to O_2_, resulting in O_2_^−^ production (Hernandez-Navarro et al., 2024). Experimental studies demonstrate that BH_4_ deficiency directly leads to eNOS-uncoupling, and restoration of BH_4_ recouples eNOS and restores NO synthesis (Crabtree et al., 2013; Li and Forstermann, 2013; De Pascali et al., 2014; Munzel et al., 2017; Channon, 2020); (b) Post-translational modifications: phosphorylation at specific threonine or tyrosine residues and other protein-protein interactions can modulate eNOS activity and coupling status (Luo et al., 2014; Munzel and Daiber, 2023); S-glutathionylation of specific cysteine residues in the eNOS reductase domain under oxidative stress acts as a redox switch that uncouples electron transfer, leading to impaired NO synthesis and increased superoxide (O_2_•^−^) generation. Experimental models show that S-glutathionylation diminishes NO output and increases superoxide production, and that mutation of these cysteine residues prevents eNOS-uncoupling (Zweier et al., 2011; Crabtree et al., 2013; De Pascali et al., 2014). (c) Disruption of eNOS dimerization: eNOS functions as a dimer, and oxidative stress or BH_4_ deficiency can disrupt dimerization and promote monomerization of eNOS, which is associated with uncoupling (Yang et al., 2009; Lee et al., 2017; Hernandez-Navarro et al., 2024). Despite these findings, there is a report suggesting that eNOS-uncoupling primarily occurs at the heme site and is not strictly dependent on monomerization (Gebhart et al., 2019); (d) Moreover, L-arginine deficiency and/or accumulation of endogenous inhibitors such as asymmetric dimethylarginine (ADMA) impairs substrate availability, favouring O_2_^.-^ production (Luo et al., 2014; Munzel and Daiber, 2023). It has been reported that L-arginine plasma concentration is lowered in aging rats (Moretto et al., 2017) and a relative I-arginine deficiency in elderly human populations, which may contribute to the endothelial dysfunction in aging (Luneburg et al., 2011; van der Zwan et al., 2013).

Since L-arginine is the only precursor of NO production through endothelial eNOS (Szlas et al., 2022) and L-arginine deficiency causes eNOS-uncoupling and reduces NO bioavailability, it was logical to implement L-arginine supplementation as adjunctive therapy to boost NO production by eNOS and to improve cardiovascular health or functions, particularly, in elderly populations in which either decreased plasma L-arginine concentration and *de novo* L-arginine synthesis or a relative deficiency exist (Luneburg et al., 2011; Kim et al., 2015; Moretto et al., 2017; Fleszar et al., 2019). However, the results are controversial, and even harmful effects have been observed and reported. In the following sections, we will analyse and discuss the results and outcomes of L-arginine supplementation and the possible underlying mechanisms. This is an important issue, since L-arginine is widely used as a supplement to boost muscle mass in healthy, diseased, and elderly populations (Flakoll et al., 2004; Borsheim et al., 2008; Deutz et al., 2013; McNeal et al., 2016).

## 3 Effects of L-arginine supplementation on cardiovascular aging and disease

L-arginine supplementation was widely implemented as an adjunctive therapy to improve endothelial function in various experimental and clinical settings (Wu et al., 2021; Saifaddin et al., 2025). Supplementation of L-arginine to isolated blood vessels from animals or humans causes vascular relaxation via enhanced endothelial NO release *in vitro* and multiple studies demonstrate that infusion of L-arginine *in vivo* causes vasorelaxation or increases blood flow in both animals and humans (Creager et al., 1992; Bode-Boger et al., 1996; Mehta et al., 1996; Joshi et al., 2007; Chuman et al., 2017). Studies in rats show that L-arginine infusion induces a dose-dependent decrease in mean arterial blood pressure and systemic vascular resistance, with increased cardiac output and stroke volume. These effects are partially NO-dependent, as shown by attenuation with NOS inhibition (Guridi et al., 2014). In healthy human subjects, intravenous L-arginine infusion (e.g., 30 g over 60 min) has been shown to significantly decrease blood pressure and total peripheral resistance, indicating vasorelaxation, and to increase blood flow in peripheral vessels. These effects are closely correlated with plasma L-arginine concentrations and are accompanied by increased urinary nitrate and cyclic GMP excretion, supporting a NO-dependent mechanism (Smulders et al., 1997; Bode-Boger et al., 1998). Intra-arterial L-arginine application also increases forearm blood flow in healthy humans in a dose-dependent manner, with no effect seen with D-arginine, further supporting the specificity of the effect (Imaizumi et al., 1992). Additionally, L-arginine infusion increases cerebral blood flow in humans, as measured by positron emission tomography, further supporting its vasodilatory properties *in vivo* (Reutens et al., 1997). In patients with peripheral vascular disease or critical limb ischemia, intravenous L-arginine infusion increases femoral artery blood flow and muscular blood flow of the calf, with effects paralleling with increased NO and cGMP production (Bode-Boger et al., 1996; Schellong et al., 1997). In patients with peripheral arterial disease, intra-arterial L-arginine acutely increases limb volumetric flow and vessel area, even in the presence of atherosclerosis (Kashyap et al., 2017). Thus, L-arginine infusion *in vivo* reliably induces vasorelaxation and increases blood flow in both healthy humans and animals, as well as in patients with vascular disease, primarily via NO-dependent mechanisms.

These beneficial effects of improving endothelial function by L-arginine supplementation, however, could not be translated to the clinical end points in patients with cardiovascular diseases. Clinical studies could not demonstrate consistent beneficial effects on endpoint outcome in cardiovascular aging or diseases, and even harmful effects are demonstrated in randomized controlled clinical trials in patients with cardiovascular diseases. The largest prospective trial in post-myocardial infarction patients (VINTAGE MI) found no improvement in vascular stiffness or ventricular remodelling with L-arginine and reported higher mortality in the L-arginine group as compared to the placebo group (Schulman et al., 2006). The trial was stopped after 6 months of the initiation. Similarly, in patients with peripheral artery diseases, L-arginine supplementation decreases NO production and shortened patients’ walking distance as compared to the placebo group (Wilson et al., 2007). Despite the negative results from these clinical studies, many studies with L-arginine supplementation have continued in either animals or humans. The results are not consistent. Meta-analyses and systematic reviews indicate that L-arginine supplementation, particularly the long-term treatment does not significantly improve endothelial function or markers of NO bioactivity in patients with cardiovascular or metabolic disorders and kidney diseases (Rodrigues-Krause et al., 2019; Mikolajetz et al., 2026).

For patients undergoing coronary artery bypass grafting, L-arginine reduced troponin T and IL-6, indicating attenuation of myocardial injury and inflammation (Mohammadi et al., 2024). Just to mention that L-arginine supplementation does not significantly improve lipid profiles. Multiple meta-analyses of randomized controlled trials consistently show no significant effect on total cholesterol, LDL, or HDL, with only a modest reduction in triglycerides (approximately 6–7 mg/dL) that is of uncertain clinical significance in healthy populations (Hadi et al., 2019; Sepandi et al., 2019). Regarding inflammatory markers, L-arginine supplementation does not significantly affect CRP, TNFα, or IL-6 in the general adult population. Subgroup analyses suggest a possible increase in CRP in older adults (>60 years), those with elevated baseline CRP, or patients with cancer, but not in healthy adults (Nazarian et al., 2019; Sepandi et al., 2019; Micker et al., 2025). These results indicate that the effects of L-arginine supplementation on lipid profiles and inflammation markers are minimal and not clinically meaningful. Therefore, L-arginine shall be used with caution.

The perspectives of amino acid supplementation including L-arginine supplementation in clinics for cardiovascular health are deeply analysed and reviewed most recently (Mikolajetz et al., 2026). The underlying mechanisms of detrimental effects of L-arginine supplementation are still obscure. We will in the next sections discuss several potential mechanisms for the harmful effects of L-arginine supplementation that are proposed and investigated.

## 4 Potential mechanisms of the harmful effects of L-arginine supplementation

### 4.1 Are the harmful effects related to dosages, treatment duration, and patient status?

As with most bioactive compounds, both the efficacy and potential adverse effects of L-arginine supplementation are strongly dose- and duration-dependent. Meta-analyses show that L-arginine supplementation moderately lowers systolic and diastolic blood pressure regardless of baseline health status and both sexes, but the effect is attenuated at higher doses (>9 g/day), longer durations (>24 days), or in obese individuals (Dong et al., 2011; Shiraseb et al., 2022). Safety data indicate that single doses up to 7.5 g are generally well tolerated, without a significant increase in gastrointestinal symptoms (Shiraseb et al., 2022). Overall, these findings suggest that while moderate short-term supplementation appears safe and can provide modest cardiovascular benefits, higher doses or prolonged intake may diminish efficacy and increase the likelihood of adverse metabolic or vascular responses, underscoring the importance of careful dosing considerations.

Consistent with the dose and duration dependence, the physiological effects of L-arginine also diverge markedly between acute and chronic exposure. Short-term (acute) L-arginine administration can transiently increase plasma arginine and NO levels but does not produce sustained changes in vascular or metabolic parameters in healthy individuals (Apolzan et al., 2022). Results of long-term supplementation (weeks to months) are variable and inconsistent, perhaps depending on baseline arginine status and/or the presence of elevated endogenous eNOS inhibitor ADMA (Rodrigues-Krause et al., 2019). In patients with multiple cardiovascular risk factors, long-term supplementation (e.g., 6 months) can improve large artery elasticity and reduce systolic blood pressure and aldosterone, suggesting vascular benefit in those with baseline endothelial dysfunction (Guttman et al., 2010). In heart failure, a randomized trial demonstrated improved cardiac function and quality of life with 3 g/day for 10 weeks (Salmani et al., 2021). Long-term L-arginine supplementation may benefit some patients with endothelial dysfunction. However, patients with acute myocardial infarction or with advanced peripheral arterial disease receiving 9 g/day orally up to 6 months showed harmful effects, i.e., increased mortality and shortened walking distance associated with decreased NO production (Schulman et al., 2006; Wilson et al., 2007), while short term use up to 60 days at lower dosage 6 g/day show mild benefits in oxidative balance and pain-free walking distance (Micker et al., 2025). In patients with stable coronary artery disease and chronic angina, L-arginine at dosage 3–9 g/day for up to 3 months shows no effects on cardiovascular functions but acceptable safety (Blum et al., 2000).

The time-dependent detrimental effects of L-arginine supplementation could be shown in cell culture model and in animal models. In cultured endothelial cells, acute exposure of the cells to L-arginine leads to increased NO production, while long-term exposure of the cells to L-arginine for several days exacerbates eNOS-uncoupling, shifting eNOS activity from NO synthesis to superoxide production (Xiong et al., 2014). In aging mouse model, chronic L-arginine supplementation for 16 weeks, causes potential renal harm and accelerated vascular functional decline (Huang et al., 2020). In the SHR rats, long-term treatment with L-arginine exacerbates age-related cardiac hypertrophy, fibrosis, and did not prevent contractile dysfunction or the development of heart failure in aging SHR (Brooks et al., 2009). Moreover, it has been shown that long-term oral administration of L-arginine did not improve hemodynamic function in rats with heart failure induced by coronary artery ligation (Feng et al., 1999) and even depresses left ventricular systolic function *in vivo* in rats with aortic stenosis (Bartunek et al., 1998).

As above discussed, patients with documented endothelial dysfunction and elevated ADMA levels may benefit from L-arginine supplementation in the context of cardiovascular disease (Bode-Boger et al., 2007). In these individuals, L-arginine can restore the L-arginine/ADMA ratio, normalize endothelial function, and potentially improve vascular outcomes (Bode-Boger et al., 2007). This benefit is not observed in patients with normal ADMA levels or normal arginine metabolism (Boger et al., 2007). However, there is no evidence from randomized controlled trials showing that L-arginine supplementation in patients with high ADMA reduces cardiovascular events or mortality. Therefore, the inconsistent results of L-arginine supplementation seem related to the dosages, supplementation time duration, and patient status.

Although the underlying mechanisms of adverse effects of long-term L-arginine supplementation are not clear, there are some hints that suggest a role of enzymes involved in L-arginine metabolism and/or signalling pathways linked to inflammation, cell proliferation, and cellular senescence. We will discuss these aspects in the following sections.

### 4.2 Are the harmful effects related to arginase?

#### 4.2.1 Arginase as a central regulator of L-arginine metabolism

**FIGURE 3:**
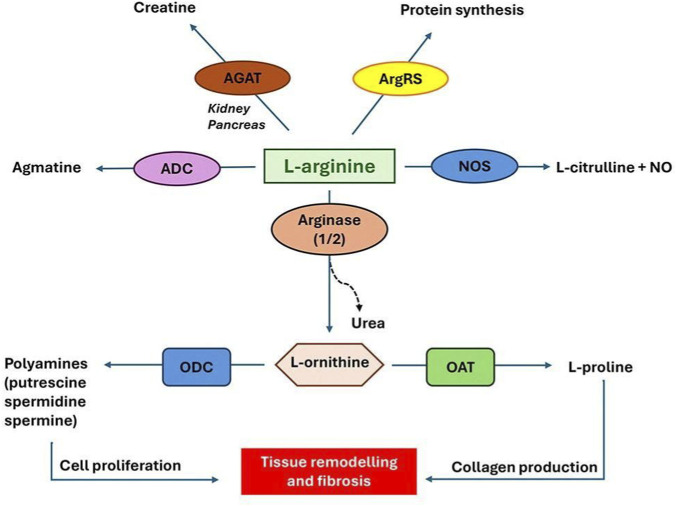
Overview of L-arginine metabolism pathways. L-arginine is metabolised via several pathways. AGAT (arginine:glycine amidinotransferase) in kidney and pancreas metabolises L-arginine to creatine; ArgRS (arginyl-tRNA synthetase) incorporates L-arginine into nascent proteins; ADC (arginine decarboxylase) converts L-arginine to agmatine; and NOS (nitric oxide synthase) produces NO and L-citrulline. Arginase 1 and arginase 2 convert L-arginine into urea and L-ornithine. L-ornithine is metabolized by ODC (ornithine decarboxylase) to generate polyamines, and by OAT (ornithine aminotransferase) to produce L-proline, a precursor for collagen synthesis.

Beyond NOS, L-arginine serves as a substrate for several other enzymes including arginase, arginine decarboxylase, and arginine:glycine amidinotransferase (AGAT) (Popolo et al., 2014; Morris, 2016; Grzywa et al., 2020; Clemente et al., 2020). Among these pathways, arginase has attracted increasing attention in the cardiovascular field because of its unique metabolic and functional roles (Figure 3). In mammalian, two isoenzymes encoded by different genes exist (Morris, 2016). Both isoforms share similar functions in metabolising L-arginine but are localized in different subcellular compartments. While Arg1 is in cytoplasm, Arg2 is in the mitochondria (Heuser et al., 2025b). Arg1 is highly expressed in the liver and hydrolyses L-arginine to L-ornithine and urea, completing the final step of the urea cycle in humans (Morris, 2016; Caldwell et al., 2018; Heuser et al., 2025b). This reaction is essential for detoxifying ammonia by converting it to urea for renal excretion. In contrast to Arg1, Arg2 is highly expressed in the renal proximal tubular cells in the S3 segment (Huang et al., 2021) and the functions of Arg2 is less clear compared to the Arg1 in the liver. Nevertheless, extrahepatic Arg2 seems more important for generating ornithine, a precursor for polyamines and proline, which are critical for cell proliferation, collagen synthesis, and tissue repair (Caldwell et al., 2018; Li et al., 2022; Heuser et al., 2025b). Thus, Arg1 remains central to hepatic nitrogen disposal, whereas Arg2 is the one predominantly implicated in cardiovascular and immune-related metabolic remodelling (Morris, 2009; Caldwell et al., 2018; Grzywa et al., 2020; Heuser et al., 2025b; Molaro et al., 2025).

Functionally, arginases, in the cardiovascular system, compete directly with eNOS for their common substrate, L-arginine. Increased arginase activity reduces substrate availability for NO production, thereby impairing endothelial function and vascular homeostasis (Durante et al., 2007; Caldwell et al., 2018). This competition promotes endothelial dysfunction, vascular remodelling, hypertension, and cardiovascular aging (Santhanam et al., 2008; Xiong et al., 2013). In addition, Arg2 exerts effects beyond substrate competition. Enhanced Arg2 expression has been mechanistically linked to mitochondrial dysfunction, increased ROS production, inflammatory activation, and vascular smooth muscle cell senescence, in part through p66Shc, p53, and redox-sensitive signalling pathways (Shin et al., 2012; Pandey et al., 2013; Xiong et al., 2013). These findings position Arg2 at a critical intersection between L-arginine utilization, mitochondrial redox balance, and inflammatory signalling.

#### 4.2.2 Species differences in arginase isoform expression

Arginase expression shows significant species- and tissue-specific differences that are critical for interpreting cardiovascular research and L-arginine supplementation studies. Across rodent and human tissues, cytosolic Arg1 is predominantly expressed in the liver involved in urea cycle, whereas mitochondrial Arg2 is highly and constitutively expressed the renal S3 segment proximal tubule epithelial cells, wherein its physiological function is not clear, yet. Arg2 is inducible in many cell types of organs and/or tissues under disease conditions and in aging, i.e., in the vascular cells and immune cells (Choi et al., 2012; Caldwell et al., 2018). Importantly, the relative contribution of each isoform to cardiovascular disease and aging differs across species. In the cardiovascular system, Arg2 is the predominant inducible isoform in mice and humans, whereas Arg1 dominates in rats (Ming et al., 2004; Yang and Ming, 2013). It has been shown that Arg1 is the predominant isoform expressed in the vascular endothelium of rat aorta, and its expression and activity are significantly upregulated with aging. Knockdown or inhibition of Arg1 restores nitric oxide (NO) signaling, improves endothelial-dependent vasorelaxation, and reduces vascular stiffness in aged rats, indicating its central role in age-related vascular dysfunction (White et al., 2006; Santhanam et al., 2007; Kim et al., 2009). Although it has been reported that both Arg1 and Arg2 are involved in cardiovascular diseases and aging (Santhanam et al., 2008; Ming et al., 2012; Caldwell et al., 2018; Zhou et al., 2018; Potenza et al., 2025), a more pronounced regulation of Arg2 under cardiovascular disease and aging is observed and genetic knockout of Arg2 gene in mouse confirms the prominent role of Arg2 in endothelial dysfunction, vascular diseases and in cardiac aging (Potenza et al., 2005; Shin et al., 2012; Yepuri et al., 2012; Xiong et al., 2013; Wu et al., 2015). Silencing Arg2 in human endothelial cells suppresses aging markers, inflammation, and restores NO bioavailability, while Arg2 knockout mice are protected from age-associated vascular dysfunction and remodelling (Ming et al., 2012; Yepuri et al., 2012). Clinical studies in elderly humans demonstrate that arginase inhibition improves endothelial function (Mahdi et al., 2019). Unfortunately, no clinical data are available to distinguish between Arg1 and Arg2.

At the cellular levels, identification of cell types that express arginase protein is crucial to understand the role of arginase in pathogenic mechanisms of cardiovascular diseases and aging phenotype and perhaps for understanding the due effects of L-arginine supplementation. Earlier studies suggested that Arg2 was present in cardiac myocytes (Khan et al., 2012); however, more recent cell-resolved and genetic approaches indicate that in both mice and humans, Arg2 is predominantly expressed in non-myocyte populations such as macrophages, fibroblasts, and endothelial cells, indicating that Arg2-driven inflammatory signaling during aging originates primarily from these non-myocyte compartments (Potenza et al., 2025), where its mitochondrial localization enables regulation of NO signaling, redox balance, and vascular tone (Ming et al., 2012; Koo et al., 2019). In contrast, rats exhibit a markedly different arginase profile: rat aortic smooth muscle cells display substantial arginase activity driven predominantly by Arg1, while Arg2 expression is low and largely confined to select vascular or immune cell subsets (Wei et al., 2001; Durante, 2013). This finding highlights a fundamental species difference.

It is interesting to note that besides the liver, Arg1 is also constitutively abundant in red blood cells (RBCs) in humans, but at very low levels in rodents (Spector et al., 1985; Heuser et al., 2025a). Functionally, RBC Arg1 is involved in regulating circulating L-arginine and NO bioavailability under both physiological and pathological conditions in human but not in mouse (Yang et al., 2013). Therefore, to study the role of RBC arginase (Arg1 and/or Arg2) in human cardiovascular disease and aging, rodent models may not be appropriate.

Thus, the interspecies differences may have major translational implications for L-arginine studies. Preclinical findings from rat models, in which Arg1 dominates, may overestimate proliferative or vasodilatory effects of L-arginine and fail to capture the mitochondrial Arg2-dependent mechanisms relevant to human cardiovascular physiology. As results, studies in rat models may be less predictive of clinical outcomes in humans, especially regarding endothelial function and aging-related vascular inflammation. By contrast, mouse models may be more closely reflect human Arg2 expression patterns in vascular and immune cells, making them more suitable for studies examining L-arginine supplementation, vascular remodeling, and Arg2-mediated inflammatory signaling. Humanized or cell-specific genetic mouse models that recapitulate human arginase distribution could provide the most translationally relevant preclinical platform to investigate therapeutic strategies targeting L-arginine metabolism.

#### 4.2.3 Arginase in cardiovascular aging and disease

Increase in arginase expression and/or activity, including Arg1 and Arg2, has been consistently observed in aging vasculature, where it contributes to impaired endothelium-dependent vasodilation, increased vascular stiffness, and enhanced susceptibility to cardiovascular diseases and dysfunction in animal models and humans (Tang et al., 2009; Xiong et al., 2014; Caldwell et al., 2015; Caldwell et al., 2018; Huang et al., 2020). Aging-associated oxidative and inflammatory environments further amplify arginase activity through post-translational modifications, most notably S-nitrosylation, which enhances enzymatic function and intensifies competition with NOS for L-arginine (Santhanam et al., 2007; Dunn et al., 2011). This age-related increase in arginase may mirror the context-dependent shifts in L-arginine utilisation, where arginase becomes a dominant competitor for eNOS under inflammatory or stress conditions (Caldwell et al., 2015; Potenza et al., 2025). By diverting L-arginine toward ornithine, polyamines and proline, elevated arginase promotes endothelial dysfunction, vascular smooth muscle cell proliferation, collagen synthesis, extracellular matrix deposition, and fibrosis, all of which are essential contributors to arterial stiffening and maladaptive cardiovascular remodelling in aging and cardiovascular diseases (please see Figure 3). This diversion of substrate away from eNOS could particularly be detrimental in diseases where immune activation or oxidative stress is present, where NOS-uncoupling and ROS production are already elevated, creating a feed-forward loop that exacerbates endothelial injury. Consistent with this concept, pharmacological inhibition or genetic ablation of arginase improves endothelial function, vascular architecture and systemic L-arginine bioavailability in animal models and elderly humans, underscoring arginase as a promising therapeutic target for age-related cardiovascular disease (Tang et al., 2009; Ming et al., 2012; Yepuri et al., 2012; Caldwell et al., 2018).

In animal models and/or cell culture models, enhanced Arg2 expression or activity has been mechanistically linked to cardiovascular dysfunction through both enzymatic and non-enzymatic or NOS-independent mechanisms (Yepuri et al., 2012; Pandey et al., 2013; Xiong et al., 2013; Heuser et al., 2025b; Potenza et al., 2025). Specifically, Arg2, beyond its canonical urea-hydrolase activity, influences mitochondrial redox balance, p66Shc-dependent stress signalling, p53 activation, and cellular senescence, thereby contributing to plaque vulnerability, endothelial apoptosis and vascular aging (Xiong et al., 2013). Arg2-driven mitochondrial dysfunction and oxidative stress amplify endothelial damage and promote eNOS-uncoupling, further reducing NO bioavailability (Shin et al., 2012; Yepuri et al., 2012). This result demonstrates a critical role of Arg2 in regulation of L-arginine utilisation, mitochondrial dysfunction, redox regulation, and inflammatory signalling in development of cardiovascular diseases and aging in mouse models. Since arginase directly competes with eNOS for L-arginine and diverts the substrate toward ornithine and polyamine synthesis, pathways linked with collagen synthesis and vascular remodelling, tissue fibrosis and cellular senescence (Durante et al., 2007; Wetzel et al., 2020), arginase could be of relevance, when considering the context-dependent effects of L-arginine supplementation, especially under chronic inflammatory conditions where arginase, particularly Arg2 becomes preferentially induced (Ming et al., 2004; Ming et al., 2012; Xiong et al., 2014).

#### 4.2.4 Is arginase involved in the detrimental effect of L-arginine supplementation?

Although acute L-arginine supplementation enhances endothelial NO production, prolonged exposure produces the opposite effect. In endothelial cells, short-term L-arginine increases NO, whereas long-term treatment reduces NO and increases superoxide due to eNOS-uncoupling, accompanied by Arg2 upregulation, S6K1 activation, and increased adhesion molecule expression in cultured human endothelial cells (Xiong et al., 2014). Silencing Arg2 or inhibiting S6K1 prevents these effects, demonstrating that Arg2-S6K1 signalling is a central driver of endothelial dysfunction as demonstrated in vascular aging in mouse models (Yepuri et al., 2012). Furthermore, animal studies show that chronic L-arginine supplementation can have deleterious effects, particularly in aging mice, such as exacerbating albuminuria and increased mortality, which are associated with elevated Arg2 levels and oxidative stress, indicating arginase-mediated renal injury, particularly in female mice (Huang et al., 2020). By limiting substrate for eNOS, heightened arginase activity reinforces the pro-fibrotic and proliferative changes, the characteristics of vascular and renal aging (Berkowitz et al., 2003; Durante et al., 2007; Santhanam et al., 2008; Shin et al., 2012; Xiong et al., 2013; Wetzel et al., 2020). These mechanisms likely contribute to the failure of L-arginine supplementation to improve cardiovascular outcomes, and in some cases, to its worsening of disease progression (Beckman et al., 2018). Efforts have been made to investigate the link of adverse effects of L-arginine supplementation to arginase in cardiovascular or renal functions in animal models. Studies in rats report decreased arginase activities in plasma and specific tissues such as the intestine and aorta, and the effects are dependent on dose and duration of L-arginine supplementation (Moretto et al., 2017; Martin and Desai, 2020; Kim et al., 2023). However, in aging mouse model, chronic L-arginine supplementation increases renal Arg2 levels, exacerbates albuminuria, and accelerates functional decline of the kidney and causes endothelial dysfunction, i.e., increased ROS and decreased NO production. These detrimental effects are at least partly prevented by Arg2 gene knockout (Huang et al., 2020). Moreover, L-arginine supplementation group reveals higher mortality as compared to the control group, particularly in female animals (Huang et al., 2020). Chronic L-arginine supplementation increases mortality and exacerbates age related kidney/vascular pathology preferentially in female mice, likely due to sex differences in Arg2 expression and NO regulation (Xiong et al., 2017a; Huang et al., 2020; Mohammad et al., 2020). It is interesting to note that female mice in aging exhibit much higher Arg2 levels in several organs, including heart (Xiong et al., 2017a; Potenza et al., 2025), lung (Zhu et al., 2023), kidneys (Huang et al., 2021), and pancreas (Xiong et al., 2017b). These findings may explain the more pronounced aging phenotype of the organs and stronger anti-aging effects of *Arg2* knockout in the females than males during the natural aging (Xiong et al., 2017a; Xiong et al., 2017b; Huang et al., 2021; Zhu et al., 2023; Potenza et al., 2025). So far, there are no human studies demonstrating sex-related difference in Arg2 expression between men and women, and whether this is linked to sex-specific cardiovascular disease and aging process is not known. Current human trials do not systematically evaluate or report sex differences in clinical responses to L-arginine supplementation. This omission can mask beneficial or harmful responses that differ by sex, limit the ability to personalize dosing or indications, undermine understanding of long-term risks in specific populations (e.g., elderly women). Future clinical research shall include sex-specific analyses to ensure both efficacy and safety profiles in men and women.

**FIGURE 4:**
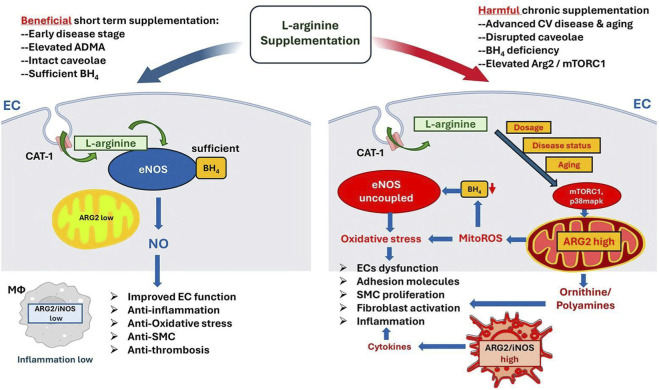
Beneficial short-term effects and harmful long-term consequences of L-arginine supplementation. Left panel: in early disease stages, short-term L-arginine supplementation enhances NO production by increasing substrate delivery through CAT-1 to eNOS, while mitochondrial Arg2 and macrophage Arg2/iNOS expression remain low under low-inflammatory conditions. Right panel: in advanced cardiovascular disease and aging, chronic high-dose L-arginine supplementation becomes detrimental. BH_4_ deficiency, elevated Arg2, and heightened mTORC1/S6K1 and p38 MAPK signalling enhance Arg2, leading to eNOS-uncoupling, mitochondrial ROS production, and inflammation, which is at least partly mediated by macrophages.

The higher mortality rate of L-arginine supplementation in the adult mouse model could be reduced by Arg2 gene knockout (Huang et al., 2020), which supports the role of Arg2 in mediating the negative outcomes of L-arginine supplementation. Furthermore, chronic exposure of human endothelial cells to L-arginine upregulates Arg2 expression and activity, which is mechanistically associated with endothelial cell senescence, increased expression of adhesion molecules, eNOS-uncoupling, and enhanced superoxide production (Xiong et al., 2014). Importantly, silencing Arg2 prevents these adverse effects, indicating a direct link between Arg2 activity and L-arginine-induced endothelial dysfunction. The underlying mechanism is shown to be due to activation of mTORC1/S6K pathway, since silencing S6K1 reduces the endothelial dysfunction caused by L-arginine in the cultured endothelial cells (Xiong et al., 2014). The mutual interaction of Arg2 and S6K1 is also demonstrated in aging mouse model (Yepuri et al., 2012). Considering that L-arginine is a potent activator of mTORC1/S6K1 in vascular endothelial cells (Yepuri et al., 2012; Xiong et al., 2014), a pathway strongly implicated in overnutrition-induced metabolic dysfunction and aging (Scalera et al., 2009; Yang and Ming, 2012) and the reciprocal interaction between Arg2 and S6K1 in promoting vascular aging (Yepuri et al., 2012), it is plausible that chronic L-arginine supplementation amplifies Arg2-mTORC1/S6K1 signalling, thereby accelerating cardiovascular aging (Figure 4). This hypothesis warrants further investigation. Future studies shall confirm whether arginase inhibition can mitigate the adverse effects of L-arginine supplementation in patients with cardiovascular or renal disease. This depends on the development of specific and efficient Arg2 inhibitors.

It is to note that small-molecule arginase inhibitors (e.g., INCB001158/CB-1158) have been developed and demonstrated acceptable safety profiles in oncology settings, but these studies did not assess vascular endpoints or cardiovascular outcomes (Naing et al., 2024). Thus, direct clinical evidence that arginase inhibition improves endothelial function or mitigates the adverse effects of L-arginine supplementation in humans is currently lacking. Indirect support comes from interventions that reduce arginase activity, such as L-citrulline supplementation, which increases NO availability and lowers arginase activity in patients with type 2 diabetes, supporting the broader concept that targeting arginase might be able to improve vascular function (Shatanawi et al., 2020). Robust clinical outcome data for pharmacological arginase inhibitors, particularly in renal or cardiovascular disease, are lacking. Notably, genetic deletion of Arg2 in mice does not prevent L-arginine-induced albuminuria, suggesting that arginase shall not be the only mechanism responsible for all adverse effects of L-arginine supplementation (Huang et al., 2020).

### 4.3 Are the harmful effects related to other cell types expressing arginase?

Beyond endothelial cells, arginase is inducible in other cell types in cardiovascular system such as macrophages, vascular smooth muscle cells, fibroblasts, etc., (Ming et al., 2012; Xiong et al., 2013; Potenza et al., 2025). Activated macrophages and neutrophils express high-output iNOS, generating large amounts of NO that rapidly react with superoxide to form peroxynitrite, driving nitrosative stress, which could enhance BH_4_ oxidation and further activate arginase, exacerbating eNOS-uncoupling in cardiovascular disease (Buttery et al., 1996; Forstermann and Munzel, 2006; Chen et al., 2010) (see Figure 4). This broader perspective becomes particularly important in pathological settings such as myocardial infarction, where acute and massive activation of the immune response, oxidative stress, and tissue remodelling dramatically alter how different cell populations compete for and utilize L-arginine. In such contexts, interaction between different cell types that utilizes L-arginine and/or express arginase will influence whether L-arginine supplementation ultimately exerts protective or detrimental effects. Studies report that macrophages express both Arg1 and Arg2 (Yang and Ming, 2014). Both isoforms are associated with different macrophage functions. While Arg1 is associated with anti-inflammatory functions of the macrophages (Yang and Ming, 2014), Arg2 has been shown to be pro-inflammatory and plays a role in cardiovascular diseases and aging (Ming et al., 2012; Yepuri et al., 2012; Huang et al., 2021; Zhu et al., 2023; Potenza et al., 2025). Mice deficient in Arg2 gene (Arg2^−/−^) are protected from inflammaging in various organs including heart, blood vessels, kidney, lung, and metabolic tissues (Ming et al., 2012; Xiong et al., 2017b; Huang et al., 2021; Zhu et al., 2023; Potenza et al., 2025). The age-associated tissue fibrosis and inflammation as well as glucose intolerance are significantly prohibited (Ming et al., 2012; Xiong et al., 2017b; Huang et al., 2021; Zhu et al., 2023; Potenza et al., 2025). Development of atherosclerosis and obesity-associated type 2 diabetes in mouse models are also reduced (Ming et al., 2012). It is however not known whether Arg2 in macrophages could be induced by L-arginine supplementation and contributes to the adverse effects of L-arginine supplementation. Moreover, fibroblasts from heart and lung also express Arg2, which is upregulated in aging. The increased Arg2 in fibroblasts enhances cytokine production and collagen production, contributing to cardiac and pulmonary fibrosis in aging (Zhu et al., 2023; Potenza et al., 2025). Whether L-arginine supplementation could upregulate arginase in fibroblasts and whether fibroblasts are a part of the game players in the adverse effects of L-arginine supplementation remain to be investigated.

### 4.4 Are the harmful effects related to the metabolic compartmentalization?

**FIGURE 5:**
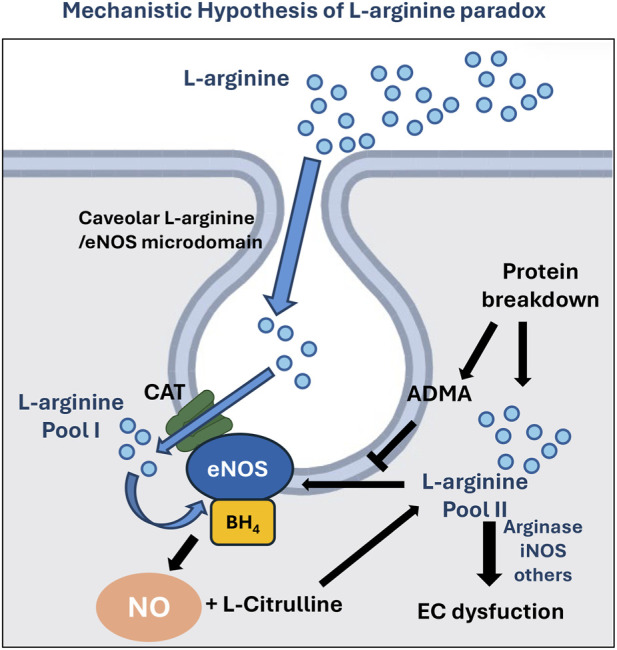
Hypothesis of L-arginine paradox. Intact caveolae form a localized L-arginine/eNOS microdomain where the cationic amino acid transporter (CAT) delivers extracellular L-arginine to eNOS for NO production. A separate intracellular L-arginine pool (pool 2) which is accessible by other enzymes including Arg2 and iNOS, which could influence endothelial NO production through decreased availability of this L-arginine pool for eNOS. Under the condition of the caveolar eNOS pathway disruption, the L-arginine supplementation is unable to improve NO production and instead enhance pool 2 functions, which could cause detrimental effects.

Although intracellular L-arginine concentrations (∼100 μM) are theoretically sufficient to saturate eNOS (Km ∼ 2–29 μM) (Hardy and May 2002), exogenous supplementation can still enhance NO production and improve endothelial function, a phenomenon known as the arginine paradox (Elms et al., 2013). This apparent contradiction is understood to arise from either the presence of endogenous eNOS inhibitor ADMA that competitively limit L-arginine availability to eNOS(Boger, 2004; Bode-Boger et al., 2007) or the metabolic compartmentalization, whereby eNOS activity is confined to specialized plasma membrane caveolar microdomains that rely on localized L-arginine transport (e.g., via CAT-1), intact caveolae structure, and adequate BH_4_ availability (McDonald et al., 1997; Chen et al., 2013) (Figure 5). In healthy or early-stage disease states, endothelial caveolar microdomains remain functionally intact, allowing supplemented L-arginine to effectively increase local substrate availability and enhance endothelial NO production. These mechanistic effects are consistent with short-term clinical studies in healthy volunteers and patients with peripheral vascular disease demonstrating improved flow-mediated dilation and vascular reactivity (Creager et al., 1992; Imaizumi et al., 1992; Bode-Boger et al., 1996; Mehta et al., 1996; Reutens et al., 1997; Schellong et al., 1997; Smulders et al., 1997; Bode-Boger et al., 1998; Kashyap et al., 2017). However, in advanced cardiovascular disease, aging, and chronic metabolic disorders, the spatial organization of L-arginine metabolism becomes impaired, particularly within endothelial caveolae. Structural alterations of caveolae, together with disrupted coupling between cationic amino acid transporters (e.g., CAT-1) and eNOS, limit the efficient delivery of extracellular L-arginine to the eNOS-associated microdomain required for NO synthesis (Kaye et al., 2000; Goligorsky et al., 2002; Potje et al., 2019; Huo et al., 2025).

In addition, studies also suggest that intracellular L-arginine is partitioned into functionally distinct pools which are poorly interchangeable (Everson and Smart, 2001; Shaul, 2002; Su, 2014) (Figure 5). In human endothelial cells, two intracellular L-arginine pools are proposed (Simon et al., 2003). The pool I represents L-arginine that is freely exchangeable with the extracellular space and is primarily supplied by extracellular L-arginine entering through membrane transporters (particularly CAT-1). This pool I provides fast and efficient substrate delivery to eNOS when extracellular arginine is available under physiological condition. In contrast, the pool II is not freely exchangeable with the extracellular space but remains accessible to eNOS. This pool is further subdivided into two components, i.e., pool IIA in which L-arginine is derived from L-citrulline recycling to sustain NO production, while the pool 2B arginine is supplied by intracellular protein degradation, whereby the protein-derived endogenous NOS inhibitors asymmetric dimethylarginine (ADMA) could be generated under pathological conditions. The pool II is accessible by other enzymes including Arg2 and iNOS, which could influence endothelial NO production through decreased availability of this L-arginine pool for eNOS (Figure 5). The L-arginine compartmentalization concept helps explain why endothelial dysfunction can occur despite high total intracellular arginine concentrations, and why the vascular benefits of L-arginine supplementation are diminished or lost in advanced disease states, when the pool I system is disrupted. In aging, circulating L-arginine levels are often maintained within the normal range, and some human cohorts have shown only modest changes or even age-related increases rather than a marked decline (Kouchiwa et al., 2012; Deutz et al., 2018). The described concept of disrupted endothelial caveolar microdomain, L-arginine compartmentation, accumulation of ADMA, increased arginase activity, could lead to a relative intracellular L-arginine deficiency in aging. However, the existence of true compartmentalized arginine pools in human endothelial cells still remains a hypothesis and debated, which requires further characterization. Moreover, the question remains open whether the L-arginine metabolizing enzyme location is the crucial mechanism for “L-arginine paradox”.

In contrast to eNOS, iNOS and Arg2 retain access to cytosolic or mitochondrial L-arginine pools, which are not dependent on caveolar integrity (Kolodziejska et al., 2005; Stefkovich and Titchenell, 2024) (Figure 5). The access of iNOS and arginase to cytosolic or mitochondrial L-arginine pools, independent of caveolar integrity, could be related to the adverse effects with L-arginine supplementation, because these enzymes utilize L-arginine from intracellular pools that are not freely exchangeable with extracellular L-arginine (Topal et al., 2006) and their activity could be upregulated by chronic supplementation, leading to increased arginase and subsequent endothelial dysfunction, oxidative stress, and cellular senescence. In another word, the compartmentalization of L-arginine pools means that L-arginine supplementation does not uniformly increase substrate availability for NO synthesis, but can instead fuel arginase activity, exacerbating adverse effects.

This mechanistic framework provides a plausible explanation for the divergence between beneficial short-term endothelial effects in healthy individuals (Creager et al., 1992; Imaizumi et al., 1992; Bode-Boger et al., 1996; Mehta et al., 1996; Reutens et al., 1997; Schellong et al., 1997; Smulders et al., 1997; Bode-Boger et al., 1998; Kashyap et al., 2017) and the neutral or harmful outcomes observed in larger clinical trials in patients with advanced cardiovascular disease, such as the increased mortality reported in post-myocardial infarction patients receiving long-term L-arginine supplementation (Schulman et al., 2006; Wilson et al., 2007). Thus, the arginine paradox is not simply a biochemical curiosity but reflects a context-dependent balance between compartmentalized NO production and maladaptive arginine metabolism, which critically determines clinical efficacy and safety. Nonetheless, this hypothesis remains incompletely validated in humans and warrants further investigation using integrated molecular and clinical approaches.

## 5 Conclusion

The effects of L-arginine supplementation and the potential underlying mechanisms are summarized in Figure 4. In short-term studies, L-arginine supplementation enhanced endothelial NO production via eNOS, leading to improved vasodilation and endothelial function. However, these mechanistic benefits do not consistently translate into improved clinical outcomes in patients with advanced cardiovascular disease or elderly populations receiving long-term supplementation. The Table 1 summarizes the major findings of randomized clinical trials of L-arginine supplementation with different dosages and duration in various patient populations since 1990 to 2026. This analysis shows that long-term L-arginine supplementation in patients showed higher mortality and worsening outcomes (Schulman et al., 2006; Wilson et al., 2007). Routine L-arginine supplementation in heart failure or general cardiovascular disease populations is not supported by robust evidence, except in the subgroup of patients with biochemically confirmed arginine deficiency or abnormal ADMA metabolism and documented endothelial dysfunction. Particularly, L-arginine supplementation should be avoided in post-myocardial infarction patients consistent with AHA guidelines (Chow et al., 2023).

**TABLE 1:** HUMAN placebo controlled Randomised studies.

Study duration	References	Population (n)	Dose and duration	Clinical outcome
Short (<4 weeks)	Bednarz et al., 2000 (int J Cardiol)	25 patients (stable CAD)	6 g/d for 3 days	Unchanged QT/ST responses during exercise, with improved exercise tolerance
	Bednarz et al., 2004 (Kardiol Pol)	21 patients (stable NYHA II-III CHF)	9 g/d for 7 days	Prolongs exercise duration; no evidence of antioxidant effects
	Ceremuzyński et al., 1997 (Am J Cardiol)	22 patients (stable angina pectoris and healed MI)	6 g/d for 3 days	Increased exercise capacity
Mid (4 to 12 weeks)	Rector et al., 1996 (Circulation)	15 patients (HF)	5.6–12.6 g/d for 6 weeks	Improves exercise capacity, vascular function, and HF functional status
	Salmani et al., 2021 (Clin Nutr)	50 patients (ischemic HF)	3 g/d for 10 weeks	Improves cardiac recovery and function, and quality of life in patients with HF
Long (>12 weeks)	Fontanive et al., 2009 (Med Sci Monit)	68 patients with systolic HF (NYHA class II-III)	2 g/d for 3 months	No significant benefit over placebo in QoL or echocardiographic parameters
	Wilson et al., 2007 (Circulation)	133 subjects with PAD	3 g/d for 6 months	No vascular effect; no improvement in walking distance
	Schulman et al., 2006 (JAMA)	153 patients after first STEMI	9 g/d for 6 months	No benefit on vascular stiffness or EF, with increased mortality versus placebo

Summary of randomized, placebo-controlled clinical trials evaluating L-arginine supplementation in adults with cardiovascular disease or elevated cardiovascular risk. Studies were identified through a structured PubMed (MEDLINE) search up to May 2026 and included randomized controlled trials with L-arginine administered as a single intervention and reporting clinical outcomes. Eligible populations comprised patients with myocardial infarction, coronary artery disease, heart failure, or peripheral artery disease, with studies involving combination therapies, non-controlled designs, or non-human data excluded. Study duration was categorized as short-term (<4 weeks), mid-term (4–12 weeks), or long-term (≥12 weeks).

Given that many elderly individuals use over-the-counter L-arginine for months or years, it is important to check the healthy status and to know the safe limit of dosage and duration of L-arginine supplementation. Based on the clinical studies discussed in the Section 4.1., the use of dosages and treatment duration of oral L-arginine supplementation shall consider the disease subtypes of patients and require strict control:

1. Avoid and contraindicated: Elderly patients with post-acute myocardial infarction; patients with advanced peripheral arterial disease (harmful effects shown with dosage 9 g/day up to 6 months).
2. Use with caution: Healthy elderly populations, stable coronary artery disease (limited benefit on optimal medical therapy); heart failure (risk of hypotension with concurrent vasodilators/nitrates), with concurrent spironolactone therapy (hyperkalaemia risk).
3. Safe upper limits by conditions: Heart failure: 3–6 g/day for ≤10 weeks; Stable angina: 3–9 g/day for ≤3 months; peripheral arterial disease: 6 g/day for ≤60 days. Patients shall be monitored for blood pressure and potassium (especially with spironolactone).

It is to emphasize that certain patient populations may still benefit from L-arginine supplementation such as patients with evidenced elevated ADMA levels and endothelial dysfunction. However, this conclusion shall be supported in the future by the hard-core clinical outcomes such as clinical developments of cardiovascular diseases and mortality by scientifically well-designed clinical trials.

## 6 Future perspectives to explore mechanisms and limit harmful effects of chronic L-arginine supplementation

**FIGURE 6:**
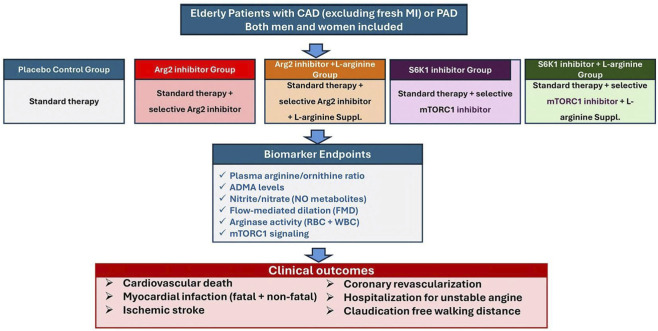
Proposed translational strategy and clinical trial design combining L-arginine supplementation with selective Arg2 or mTORC1 inhibition. The trial enrols patients with cardiovascular disease (e.g., coronary artery disease and peripheral artery disease) (fresh myocardial infarction excluded according to AHA guidelines) and randomizes them into five groups: (1) control group receiving standard-of-care therapy, (2) standard therapy + Arg2 inhibitor, (3) standard therapy + Arg2 inhibitor + L-arginine supplementation, (4) standard therapy + selective mTORC1 inhibitor, and (5) standard therapy + mTORC1 inhibitor + L-arginine supplementation. This design tests whether inhibiting Arg2 or mTORC1 alone, or in combination with L-arginine, improves cardiovascular outcomes.

Experimental evidence indicates that chronic or long-term L-arginine supplementation may have detrimental effects mediated by arginase and/or mTORC1 pathways, but these hypotheses require further investigation in appropriately designed preclinical and clinical studies. Future research should include analysis of sex-specific effects and mechanism-guided strategies to improve both the efficacy and safety of L-arginine therapy. Preclinical data suggest that older female mice are particularly susceptible to Arg2-mediated oxidative stress and vascular/renal injury, which can increase mortality. A testable hypothesis is that whether combining L-arginine supplementation with selective Arg2 or/and mTORC1 inhibition could enhance cardiovascular functions and improve clinical outcome in patients including men and women (Figure 6). Clinical endpoints such as plasma arginine/ornithine ratio, ADMA, nitrite/nitrate levels, flow-mediated dilation, arginase activities and mTORC1 signaling in RBC and WBC could serve as biomarkers to track efficacy in patients particularly elderly populations with cardiovascular disease either coronary artery disease or peripheral artery disease. The clinical outcomes such as cardiovascular death, myocardial infarction (nonfatal and fatal), stroke (ischemic), coronary revascularization, unstable angina requiring hospitalization could be analysed. Such a translational roadmap allows to prove the above discussed hypothesis.

Another interesting and relevant issue that could be involved in adverse effects of long-term oral L-arginine supplementation is the microbial metabolism of the amino acid in the gut. The impact of gut microbial metabolism of dietary nutritional components including L-arginine on cardiovascular diseases are increasingly appreciated (Nuse et al., 2023; Wu et al., 2025). Evidence demonstrates that oral L-arginine is significantly metabolized by gut microbiota before absorption, and microbial enzymes including arginase, decarboxylases, and other arginine-metabolizing enzymes can influence both systemic arginine bioavailability and production of various metabolites (Nuse et al., 2023). Gut bacteria metabolize L-arginine through multiple enzymatic pathways, including microbial arginase, which converts arginine to ornithine and urea. Moreover, microbial arginine decarboxylase produces agmatine and polyamines from L-arginine. Various metabolites including ammonia, short-chain fatty acids, and other nitrogen-containing compounds could be generated from L-arginine by gut bacteria (Abdallah et al., 2020; Nuse et al., 2023). It has been estimated that approximately 60% of dietary arginine is converted to urea during first-pass splanchnic metabolism, with kinetics indicating substantial extraction before systemic absorption (Mariotti et al., 2013). The complex interplay between host and microbial arginine metabolism, combined with adaptive upregulation of arginase, creates a scenario where acute benefits may not translate to chronic efficacy and could potentially cause harm in certain populations. This aspect warrants further investigation.

Overall, elucidating the mechanisms of the detrimental effects of chronic L-arginine supplementation remains a highly interesting research area of clinical relevance, not only for cardiovascular disease but also in the context of comorbidities such as chronic kidney disease. Understanding these pathways may identify novel, personalized therapeutic approaches to safely leverage the beneficial vascular effects of L-arginine while minimizing potential harm (Mikolajetz et al., 2026).
